# Association of Neighborhood-Level Disadvantage With Alzheimer Disease Neuropathology

**DOI:** 10.1001/jamanetworkopen.2020.7559

**Published:** 2020-06-11

**Authors:** W. Ryan Powell, William R. Buckingham, Jamie L. Larson, Leigha Vilen, Menggang Yu, M. Shahriar Salamat, Barbara B. Bendlin, Robert A. Rissman, Amy J. H. Kind

**Affiliations:** 1 Department of Medicine, Geriatrics Division, University of Wisconsin School of Medicine and Public Health, Madison; 2 Health Services and Care Research Program, University of Wisconsin School of Medicine and Public Health, Madison; 3 Department of Biostatistics & Medical Informatics, University of Wisconsin, Madison; 4 Department of Neurological Surgery, University of Wisconsin School of Medicine and Public Health, Madison; 5 Department of Pathology and Laboratory Medicine, University of Wisconsin School of Medicine and Public Health, Madison; 6 Wisconsin Alzheimer’s Disease Research Center, University of Wisconsin School of Medicine and Public Health, Madison; 7 Geriatric Research Education and Clinical Center (GRECC), William S. Middleton Hospital, United States Department of Veterans Affairs, Madison, Wisconsin; 8 Wisconsin Alzheimer’s Institute, University of Wisconsin School of Medicine and Public Health, Madison; 9 Department of Neurosciences, University of California, San Diego; 10 Shiley-Marcos Alzheimer's Disease Research Center, University of California, San Diego, La Jolla

## Abstract

**Importance:**

Social determinants of health, such as income, education, housing quality, and employment, are associated with disparities in Alzheimer disease and health generally, yet these determinants are rarely incorporated within neuropathology research.

**Objective:**

To establish the feasibility of linking neuropathology data to social determinants of health exposures using neighborhood disadvantage metrics (the validated Area Deprivation Index) and to evaluate the association between neighborhood disadvantage and Alzheimer disease–related neuropathology.

**Design, Setting, and Participants:**

This cross-sectional study consisted of decedents with a known home address who donated their brains to 1 of 2 Alzheimer disease research center brain banks in California and Wisconsin between January 1, 1990, and December 31, 2016. Neither site had preexisting social metrics available for their decedents. Neuropathologic features were obtained from each site for data collected using the standardized Neuropathology Data Set form and from autopsy reports. Data were analyzed from June 7 to October 10, 2019.

**Exposures:**

Geocoded decedent addresses linked to neighborhood disadvantage as measured by the Area Deprivation Index calculated for the year of death.

**Main Outcomes and Measures:**

Presence of Alzheimer disease neuropathology. The association between neighborhood disadvantage and Alzheimer disease neuropathology was evaluated via logistic regression, adjusting for age, sex, and year of death.

**Results:**

The sample consisted of 447 decedents (249 men [56%]; mean [SD] age, 80.3 [9.5] years; median year of death, 2011) spanning 24 years of donation. Fewer decedents (n = 24 [5.4%]) originated from the top 20% most disadvantaged neighborhood contexts. Increasing neighborhood disadvantage was associated with an 8.1% increase in the odds of Alzheimer disease neuropathology for every decile change on the Area Deprivation Index (adjusted odds ratio, 1.08; 95% CI, 1.07-1.09). As such, living in the most disadvantaged neighborhood decile was associated with a 2.18 increased odds of Alzheimer disease neuropathology (adjusted odds ratio, 2.18; 95% CI, 1.99-2.39).

**Conclusions and Relevance:**

The findings of this cross-sectional study suggest that social determinants of health data can be linked to preexisting autopsy samples as a means to study sociobiological mechanisms involved in neuropathology. This novel technique has the potential to be applied to any brain bank within the United States. To our knowledge, this is the first time Alzheimer disease neuropathology has been associated with neighborhood disadvantage.

## Key Points

**Question:**

Can neighborhood disadvantage, a social determinant of health, be incorporated into existing brain bank data to evaluate the risk of biological outcomes, such as Alzheimer disease neuropathology?

**Findings:**

In this cross-sectional study using autopsy samples from 447 decedents, living in a disadvantaged neighborhood at the time of death was associated with an increased risk of presence of Alzheimer disease neuropathology when adjusting for age, sex, and year of death.

**Meaning:**

These findings suggest that neighborhood disadvantage can be geolinked to brain bank repositories and may be a marker for Alzheimer disease neuropathology.

## Teaser

This cross-sectional study assesses the feasibility of linking neuropathology data to social determinants of health by evaluating the association between neighborhood disadvantage and Alzheimer disease–related neuropathology among decedents in Wisconsin and California.

## Introduction

When it comes to health, context matters.^1,2^ Social determinants of health (SDOH) are the “conditions in the environments in which people are born, live, learn, work, play, worship, and age that affect a wide range of health, functioning, and quality-of-life outcomes and risks.”^3^ They are fundamental mechanisms within frameworks of health and health disparities,^3,4,5,6^ yet practical means to connect biological end points to contextual factors are not widely available, particularly for a majority of existing biobanks. The few biobanks that are linked to longstanding cohort studies are a notable exception. These biobanks have contributed greatly to knowledge about associations between social factors and biological outcomes but are too few in number.

This situation is now changing. With recent advances in precision public health, it is possible to capture SDOH at more granular geographic levels (eg, neighborhood conditions), improving the ability to understand how interactions between contextual and biological mechanisms drive health.^7^ Neighborhood disadvantage metrics, which reflect underlying SDOH of a precise geographic region, are now widely available through the National Institutes of Health (NIH)–funded Neighborhood Atlas.^7,8^ Furthermore, the NIH is encouraging mechanistic-focused health disparities research through targeted funding announcements and frameworks, such as the National Institute on Aging health disparities framework.^9^

Within the field of Alzheimer disease (AD) and related dementias, health disparities disproportionally affect minority and socially disadvantaged populations.^10,11,12,13,14,15^ Little is known about the fundamental biological processes that drive these associations, and few existing AD studies link brain tissue neuropathology to SDOH metrics. If more widely available, such linkages could allow for new insight into the extent to which SDOH may affect risk for AD dementia and neuropathology. These links may also unlock new vantage points for fundamental mechanistic discovery and therapeutic development.

Precision medicine necessitates not only a characterization of unique tissue and genomic factors but also an understanding of how context—environmental, sociocultural, and behavioral factors—combines with biological factors to affect our health.^16,17,18^ Precision medicine holds the promise of identifying individuals at increased risk in an effort to provide tailored treatment and prevention efforts to improve health.^18^ But this promise may not be fully realized if tissue is considered apart from context; both must be considered as part of the whole.

The first step in realizing this goal is to prove the feasibility of linking SDOH to biological data where these SDOH were not previously measured. With this in mind, the objective of this study is to employ precision public health data technologies to characterize SDOH exposures for existing brain tissue from biobanks (eFigure in the Supplement). Specifically, we examine the extent to which neighborhood disadvantage exposure is associated with AD neuropathology. By doing so, we aim to provide an illustrative example of how to determine the association between SDOH contextual risk factors and health and show the feasibility of a novel method that could potentially be applied to any biorepository in the United States and its territories. Because geographic accessibility is a key factor driving access to many research resources,^19^ the work presented here also enables us to examine the geographic alignment of the Alzheimer Disease Research Center (ADRC) brain bank network as a whole with the geographic location of disadvantaged US neighborhoods using geosimulation.

## Methods

### Study Sample

This study is a retrospective, cross-sectional analysis of decedents who donated their brains to 1 of 2 ADRC brain bank repositories. Decedents were included if they died between January 1, 1990, and December 31, 2016, had a noninstitutional address, and had complete information on the presence of diffuse and neuritic plaques (n = 447) as determined by a neuropathologist. This study was reviewed by the University of Wisconsin and University of California San Diego institutional review boards and was determined to be not human subjects research; as such, informed consent was not required. Data use agreements between the 2 sites complied with protected health information under HIPAA regulations. The Strengthening the Reporting of Observational Studies in Epidemiology (STROBE) reporting guideline was followed.

### Precision Public Health Metrics

Social determinants of health were measured by the Area Deprivation Index (ADI) determined at the neighborhood-level.^7^ The ADI is a validated, neighborhood-level composite index reflecting 17 SDOH dimensions captured in the American Community Survey and US Census Survey data via principal components analysis methodology. The resulting factor score is then transformed into a percentile ranking, facilitating comparisons across time to prior and future ADI builds and to external measures of socioeconomic disadvantage. The ADI state rankings range from 1 to 10, with least disadvantaged neighborhood conditions designated by lower scores and most disadvantaged by higher scores. Examples of neighborhood-level SDOH factors incorporated within the ADI include education level, income, housing, and employment characteristics. For this study, the ADI state decile ranking denoting the decedent’s neighborhood SDOH conditions associated with their last known residence was used. For deaths from 1990 through 2009, the ADI is based on that decade’s Decennial Census data (eg, the ADI ranking for someone who died during the 1990s is built from the 1990 Census). For 2010 and after, additional forms of Census data are available, so yearly versions of the ADI could be generated using the same dimensions via the Census’ American Community Survey.

The decedent’s address at the time of death was linked to its corresponding Census Block Group and ADI value via geoanalytic methods. Census block groups represent neighborhood geographic regions, containing a mean of 1500 people.^20^ Because census block groups are updated every decade, addresses were linked to the census block group for the decade in which the death occurred. All ADRC locations with brain bank donation were geocoded using ArcGIS 10.7 (Environmental Systems Research Institute). Distance between the ADRC brain bank and the decedent’s address at the time of death were calculated using network analysis via ArcGIS using ESRI Business Analyst (Environmental Systems Research Institute) data.

### Neuropathology Data

We relied on 2 methods to extract neuropathologic features. For each ADRC site, we used data collected via the Neuropathology Data Set form (a National Alzheimer Coordinating Center standardized data collection form).^21^ If Neuropathology Data Set data were not available from the site for a particular decedent (typically for the years prior to 2005), abstractions of the same elements were derived directly from digital scans of autopsy reports. An abstraction guide was generated using previous methods and based on expert knowledge from neuropathologists (including M.S.S.) at both centers.^22^ All autopsy reports were dually abstracted for the same data by 2 independent, trained abstractors (J.L.L., L.V.). Interrater reliability was strong, with the κ statistic ranging from 0.8 to 1.0 for all abstracted variables. Any discrepancies between abstractors were discussed and corrected via abstraction team consensus. The abstracted data from autopsy reports mirrored those of the derived variables in the Neuropathology Data Set data.

Utilizing an existing methodology as per Hassenstab et al^23^ and Monsell et al,^24^ presence of AD neuropathology was defined as the presence of either diffuse plaques or neuritic plaques, following the National Institute of Aging and the Alzheimer Association criteria for neuropathology plaque presence.^25^ For sensitivity analyses, we also used a stricter definition, which required the presence of both diffuse plaques and neuritic plaques.^5^ We used this stricter definition in sensitivity analyses to ensure robustness of the overall results. The use of this stricter definition did not change the overall results (see eTable 1 in the Supplement). In order to be consistent with existing studies, we used the methodology as per Hassenstab et al^23^ and Monsell et al^24^ within the analyses below.

### Brain Donation Catchment Simulation

We generated a nationwide geographic simulation of catchment-area subject pools for all ADRC brain banks using US census population data.^26^ Because geographic accessibility is a key factor driving access to many research resources,^19^ the goal of this simulation was to estimate geographic alignment of the ADRC brain bank network as a whole with the geographic location of disadvantaged US neighborhoods using geosimulation. All ADRC locations with brain bank donation were geocoded using ArcGIS. Once the locations were determined, the geographic analysis tools were applied to create a 100-mile circular radius around each ADRC—an estimate of a reasonable geographic accessible area for most typical participants interested in brain donation and consistent with the observed geo-reach of each brain bank donation program within our sample data (approximately 90% of decedents in the cross-sectional study lived within 100 miles). US Census block groups that intersect each buffer were then assessed. However, we recognize that some brain banks may have access that reaches beyond the 100-mile buffer used for this geosimulation. Also, this approach does not account for more motivated participants who are willing to engage with ADRCs at a great distance, which may occur more often in certain populations, such as frontotemporal dementia patients traveling great distances to seek diagnosis and care at world-renowned centers.^27^

### Statistical Analysis

Descriptive statistics for demographic, SDOH, and neuropathologic features were calculated and are reported in Table 1 and Table 2. To evaluate the association between SDOH (via the ADI) and neuropathology features, we employed a generalized linear model, modeling the logit of the binomial AD neuropathology outcome, with clustered robust standards errors to address site-level clustering. To address the concern of possible sparse data bias, corrected coefficients using rare event modeling methods that provide more conservative estimates of risk are reported.^28^ The key explanatory variable was the ADI state ranking with covariate adjustments for age, sex, and year of death. Odds ratios (ORs) and 95% CIs of resulting models are reported. Decedents without the necessary information to categorize AD neuropathology (n = 6) were removed from the analysis and were significantly younger (mean [SD] age, 62.3 [10.0] years [95% CI, 51.9-72.8 years] vs 80.3 [9.5] years [95% CI, 79.4-81.2 years]; *P* < .001 with independent, 2-sided *t* test at α = .05) but otherwise not different on mean ADI, sex, or year of death. Modeling was conducted using Stata/SE 15.1 (StataCorp LLC). The analysis was conducted from June 7 to October 10, 2019.

**Table 1.** Demographic Characteristics of Decedent Sample

Characteristic	No. (%)^a^		
	Overall sample (N = 447)	AD (n = 394)	No AD (n = 53)
Neighborhood disadvantage, mean (SD) decile	3.8 (2.3)	3.8 (2.4)	3.4 (2.1)
Age, mean (SD), y	80.3 (9.5)	80.6 (9.3)	78.4 (10.6)
Age group, y			
<65	23 (5)	19 (5)	4 (8)
65-69	47 (11)	38 (10)	9 (17)
70-74	41 (9)	36 (9)	5 (9)
75-79	71 (16)	65 (16)	6 (11)
80-84	95 (21)	81 (21)	14 (26)
85-89	98 (22)	90 (23)	8 (15)
≥90	72 (16)	65 (16)	7 (13)
Sex			
Female	198 (44)	177 (45)	21 (40)
Male	249 (56)	217 (55)	32 (60)
Year of death			
1990-1999	4 (1)	4 (1)	0
2000-2009	184 (41)	163 (41)	21 (40)
2010-2016	259 (58)	227 (58)	32 (60)

Abbreviation: AD, Alzheimer disease.

^a^ Percentages have been rounded and may not equal 100.

**Table 2.** Neuropathologic Features of Decedent Sample

Neuropathologic feature	Overall sample (N = 447)
AD neuropathology, No. (%)	
No	53 (12)
Yes	394 (88)
Diffuse plaque, No. (%)	
Not present	73 (16)
Present	374 (84)
Neuritic plaque, No. (%)	
Not present	69 (15)
Present	378 (85)

Abbreviation: AD, Alzheimer disease.

## Results

### Distribution of Disadvantage

**Figure 1.**
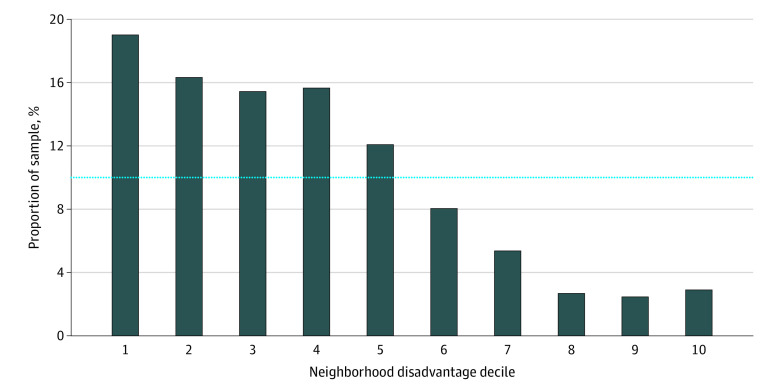
Neighborhood Disadvantage Within Decedent Sample Neighborhood disadvantage measured by Area Deprivation Index percentile rankings, ranging from 1 (least disadvantaged decile neighborhoods) to 10 (most disadvantaged decile neighborhoods). Bars denote the proportion within each neighborhood disadvantage category for the study sample. The dotted line represents the proportion within each category for the United States.

The final sample comprised autopsy data from 447 decedents (198 women [44%] and 249 men [56%]; mean [SD] age, 80.3 [9.5] years; median year of death, 2011) spanning 24 years of collection. Sociodemographic characteristics and neuropathologic features are noted in Table 1 and Table 2, respectively. The distribution of neighborhood disadvantage was right-skewed, such that most sample decedents originated from the most affluent (ie, least disadvantaged) neighborhoods, with 24 decedents (5.4%) from the most disadvantaged quintile (top 20%) neighborhoods (Figure 1). Assessing neuropathology of the sample revealed a high rate of AD neuropathology (n = 394 [88%]), which is consistent with the sample sources.

### Neighborhood Disadvantage

Decedents originating from the most disadvantaged neighborhoods had the highest risk of AD neuropathology. Adjusted model results demonstrated that with each decile increase in neighborhood disadvantage (by ADI), there was an estimated 8.1% increase in the expected odds for AD neuropathology (eTable 2 in the Supplement) (adjusted OR, 1.08; 95% CI, 1.07-1.09). As such, living in the most disadvantaged neighborhood decile was associated with 2.18 higher odds (95% CI, 1.99-2.39) of AD neuropathology. Decedents within the current study resided a median of 19 miles (interquartile range, 10-31) from their respective ADRC brain bank.

### Brain Donation Catchment Simulation

**Figure 2.**
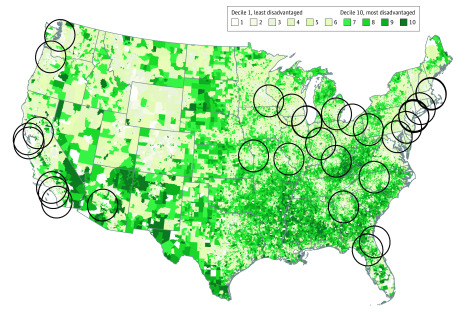
Geosimulation of Alzheimer Disease Research Center (ADRC) Catchment Areas Recruitment catchment within 100 miles of each ADRC, with darker green areas denoting neighborhoods with the highest levels of neighborhood disadvantage. The list of existing ADRCs is drawn from information listed by the National Institute on Aging.^29^

Based on geosimulation modeling, including the applied assumptions, 56% of the total US population lives within 100-miles of an ADRC^29^ and would potentially have easier geographic access to brain bank donation services (Figure 2). For the other 44% of the US population, access to ADRC brain bank donation may be more geographically difficult. Of the population within the 100-mile radius of an ADRC brain bank—ie, those with easier access—only 13% live within the top quintile of neighborhood disadvantage (the most disadvantaged neighborhoods), whereas 30% live in the most wealthy quintile (least disadvantaged) neighborhoods in the United States. This distribution of geographic access skews toward the most wealthy neighborhoods in a manner that does not reflect the general US population.

## Discussion

In this cross-sectional study, we showed the feasibility of using precision public health techniques to characterize SDOH exposures for autopsy samples in 2 ADRC brain banks. These are brain banks that did not otherwise have SDOH data linked to their biospecimens. To our knowledge, this study is the first time that precision public health approaches have been applied in this way. This novel technique has the potential to link contextual factors to autopsy tissue for any biorepository within the United States. Furthermore, as an initial use example, we used this linkage to evaluate the association between SDOH and AD neuropathology, finding that increasing neighborhood disadvantage is associated with increased odds of AD neuropathology. However, additional studies are needed to replicate this finding.

Precision medicine holds great promise to revolutionize diagnostics and therapeutics. However, biobank data should not be considered in isolation from context. A complete approach to precision medicine necessitates characterization of tissues and genes as well as employing precision public health techniques to more fully characterize contextual factors and, synergistically, to allow for a more complete study of how all factors combine to affect health. Precision public health promises “the right intervention to the right population at the right time.”^17^^(p1399)^ With an eye toward this promise, using the methods demonstrated in this study, SDOH can be linked to existing biobanks and tissue repositories as a means to catalyze the study of sociobiological mechanisms—that is, the biological mechanisms that potentially are at play in health disparities. The SDOH data within the Neighborhood Atlas that facilitate this linkage are free, easily available online, NIH supported, and accessible to all.^7^

Within this study, decedents rarely originated from the most disadvantaged neighborhoods; decedents from wealthy neighborhoods were more common. This is a pattern that likely is common within biorepositories across the United States,^30^ but additional study is needed to confirm as well as identify where representation is most lacking. Recruitment of diverse and disadvantaged participants is a known challenge within the AD research field.^31,32^ Yet improvements are actively being made by many leaders in the field.^33,34,35,36,37,38^ Some brain banks focus intensely on these populations to great success, but more science on how best to recruit and resources to fund recruitment science are needed. However, in the geosimulations conducted within the present study, even if an ADRC brain bank was able to achieve an optimally population-representative recruitment of its catchment population, the sampled population would still likely skew toward the wealthier neighborhoods. Many people within the United States still do not have easy geographic access to ADRC services, and ADRCs tend to demonstrate tight clustering within certain US regions. Additional efforts to establish ADRCs, ADRC-affiliated branch sites, or ADRC-related services within other geographic regions of the United States could be considered or explored as a potential way to increase easy geographic access to resources and to facilitate broader population samplings for research.

### Limitations

This study should be considered in light of a number of limitations. Although we utilized all available samples and employed relatively broad inclusion criteria, decedents from disadvantaged neighborhoods were not as well represented within the study sample. Selection bias may be a factor in brain donation. Therefore, these results need to be replicated in a larger, more generalizable sample, and the present interpretation and generalization must be limited. Assessments of SDOH exposure relied on place of residence at the time of death, which may not accurately reflect SDOH exposure across a life course. Additional in-depth study is underway to develop new methods to address this limitation and to provide a more robust life-course perspective to the sample. However, given the intense resource and time requirements of historical life-course assessments of brain bank decedents, these data will not be available for some time. Definitions of AD neuropathology have changed over time; however, we used a current set of criteria to mitigate this limitation to an extent.

The biosocial pathway of the association between neighborhood disadvantage and AD remains largely unclear. Much more research is needed in this area. Possible factors related to the of amyloid plaques include stress; sleep disruption; lifestyle factors, such as diet and exercise; pollution; and cardiovascular risk factors, such as diabetes.^39,40,41,42,43,44,45^ These were factors that could not be assessed within the current study, as such data were not collected on these existing brain specimens. In general, linkages to such factors within existing brain bank neuropathology resources are too few. Significant research is needed to better understand the association among these factors and outcomes and to understand how the association between ADI and AD neuropathology varies within key subgroups (eg, race/ethnicity, apolipoprotein E4).

## Conclusions

This study demonstrates how accessibility to precision public health data technologies can place brain tissue in context. This linkage offers promising ways to better elucidate the population representativeness of existing samples as well as new tools to investigate mechanisms associated with disparities and AD neuropathology.

## Notes

Supplement. **eFigure.** Putting Tissue in Context: Linking Neighborhood Social Determinants of Health to Alzheimer’s Disease Neuropathology **eTable 1.** Unadjusted and Adjusted Main and Sensitivity Model Results **eTable 2.** Unadjusted and Adjusted Results with Neighborhood Disadvantage Placed into Tertile Groupings Based on Study Sample
