# Supplementary material for: Association of Neighborhood-Level Disadvantage With Alzheimer Disease Neuropathology
Source: JAMA Netw Open. 2020 Jun 11;3(6):e207559. doi: 10.1001/jamanetworkopen.2020.7559 (PMC7290421; doi:10.1001/jamanetworkopen.2020.7559)
Supplement: Supplement. — eFigure. Putting Tissue in Context: Linking Neighborhood Social Determinants of Health to Alzheimer’s Disease Neuropathology eTable 1. Unadjusted and Adjusted Main and Sensitivity Model Results eTable 2. Unadjusted and Adjusted Results with Neighborhood Disadvantage Placed into Tertile Groupings Based on Study Sample [file jamanetwopen-3-e207559-s001.pdf]

Supplementary Online Content

Powell WR, Buckingham WR, Larson JL, et al. Association of neighborhood-level disadvantage with Alzheimer disease neuropathology. *JAMA Netw Open*. 2020;3(6):e207559. doi:10.1001/jamanetworkopen.2020.7559

eFigure. Putting Tissue in Context: Linking Neighborhood Social Determinants of Health to Alzheimer's Disease Neuropathology

eTable 1. Unadjusted and Adjusted Main and Sensitivity Model Results

eTable 2. Unadjusted and Adjusted Results with Neighborhood Disadvantage Placed into Tertile Groupings Based on Study Sample

This supplementary material has been provided by the authors to give readers additional information about their work.

© 2020 Powell WR et al. *JAMA Network Open.*

eFigure. Putting Tissue in Context: Linking Neighborhood Social Determinants of Health to Alzheimer's Disease Neuropathology

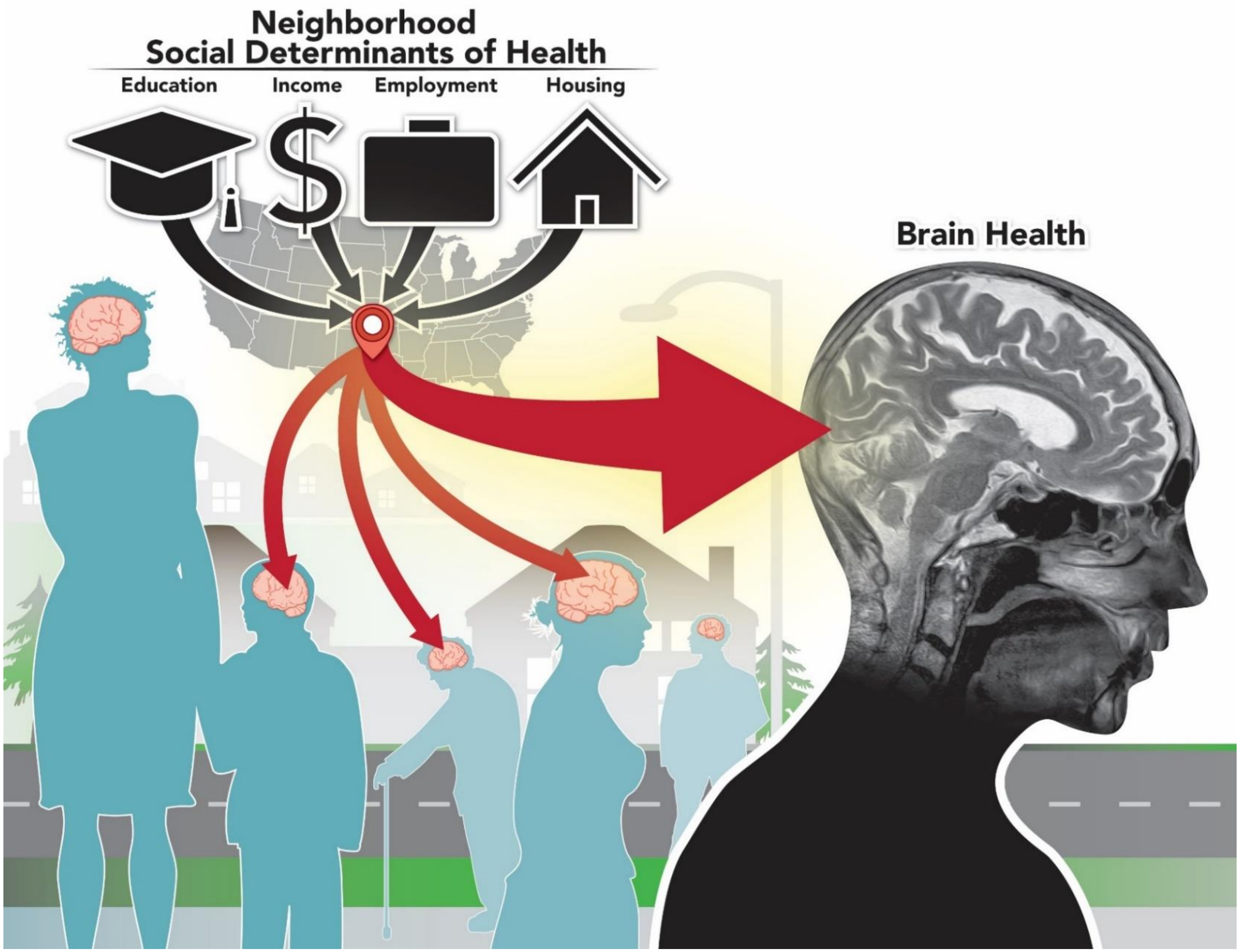

© 2020 Powell WR et al. *JAMA Network Open.*

eTable 1. Unadjusted and Adjusted Main and Sensitivity Model Results

	Main AD Neuropathology Analysis[b]				Stricter AD Neuropathology Definition[c]			
**Characteristic**	**Unadjusted OR**	**95% CI**	**Adjusted OR[a]**	**95% CI**	**Unadjusted OR**	**95% CI**	**Adjusted OR[a]**	**95% CI**
ADI neighborhood disadvantage (decile)	1.083	1.082 - 1.084	1.081	1.071 - 1.091	1.070	1.059 - 1.081	1.068	1.066 - 1.071
Age			1.021	1.014 - 1.029			1.021	1.013 - 1.030
Sex								
Female			1.000	(reference)			1.000	(reference)
Male			0.827	0.750 - 0.911			0.833	0.747 - 0.929
Year of Death			0.982	0.912 - 1.056			0.984	0.908 - 1.068

AD, Alzheimer's disease; ADI, Area Deprivation Index; CI, confidence interval; OR, odds ratio
[a] Adjusted for age, sex, and year of death
[b] AD neuropathology defined as presence of either diffuse or neuritic plaques (n=447)
[c] AD neuropathology defined as presence of both diffuse and neuritic plaques (n=415)

© 2020 Powell WR et al. *JAMA Network Open.*

eTable 2. Unadjusted and Adjusted Results with Neighborhood Disadvantage Placed into Tertile Groupings Based on Study Sample

	**Main AD Neuropathology Analysis[b]**			
**Characteristic**	**Unadjusted OR**	**95% CI**	**Adjusted OR[a]**	**95% CI**
Neighborhood disadvantage tertile groups				
Least disadvantaged (ADI deciles 1 & 2)	1.000	(reference)	1.000	(reference)
Middle disadvantaged (ADI deciles 3 & 4)	1.170	1.152 - 1.189	1.130	1.068 - 1.196
Most disadvantaged (ADI deciles 5 to 10)	1.274	1.038 - 1.564	1.262	1.103 - 1.445
Age			1.022	1.016 - 1.028
Sex				
Female			1.000	(reference)
Male			0.835	0.772 - 0.903
Year of Death			0.984	0.914 - 1.058

AD, Alzheimer's disease; ADI, Area Deprivation Index; CI, confidence interval; OR, odds ratio
[a] Adjusted for age, sex, and year of death
[b] AD neuropathology defined as presence of either diffuse plaques or neuritic plaques (n=447)

© 2020 Powell WR et al. *JAMA Network Open.*
